# Cross-cultural adaptation, validity, and reliability of the Persian version of the spine functional index

**DOI:** 10.1186/s12955-018-0928-5

**Published:** 2018-05-15

**Authors:** Hamid Reza Mokhtarinia, Azadeh Hosseini, Azam Maleki-Ghahfarokhi, Charles Philip Gabel, Majid Zohrabi

**Affiliations:** 1 Department of Ergonomics, University of Social Welfare and Rehabilitation Sciences, Kodakyar Ave.Daneshjo Blvd., Evin, Tehran, PC: 1985713834 Iran; 2 Department of Ergonomics, University of Social Welfare and Rehabilitation Sciences, Tehran, Iran; 3 Student Research Committee, Faculty of Health, Tabriz University of Medical Sciences, Tabriz, Iran; 4 Coolum Physiotherapy, Sunshine Coast, QLD Australia; 5 Department of Neurosurgery, Nikan Hospital, MD, Neurosurgeon, Tehran, Iran

**Keywords:** Spine outcome, Persian, Reliability, Validity, Factor analysis

## Abstract

**Background:**

There are various instruments and methods to evaluate spinal health and functional status. Whole-spine patient reported outcome (PRO) measures, such as the Spine Functional Index (SFI), assess the spine from the cervical to lumbo-sacral sections as a single kinetic chain. The aim of this study was to cross-culturally adapt the SFI for Persian speaking patients (SFI-Pr) and determine the psychometric properties of reliability and validity (convergent and construct) in a Persian patient population.

**Methods:**

The SFI (English) PRO was translated into Persian according to published guidelines. Consecutive symptomatic spine patients (104 female and 120 male aged between 18 and 60) were recruited from three Iranian physiotherapy centers. Test-retest reliability was performed in a sub-sample (*n* = 31) at baseline and repeated between days 3–7. Convergent validity was determined by calculating the Pearson’s r correlation coefficient between the SFI-Pr and the Persian Roland Morris Questionnaire (RMQ) for back pain patients and the Neck Disability Index (NDI) for neck patients. Internal consistency was assessed using Cronbach’s α. Exploratory Factor Analysis (EFA) used Maximum Likelihood Extraction followed by Confirmatory Factor Analysis (CFA).

**Results:**

High levels of internal consistency (α = 0.81, item range = 0.78–0.82) and test-retest reliability (*r* = 0.96, item range = 0.83–0.98) were obtained. Convergent validity was very good between the SFI and RMQ (*r* = 0.69) and good between the SFI and NDI (*r* = 0.57). The EFA from the perspective of parsimony suggests a one-factor solution that explained 26.5% of total variance. The CFA was inconclusive of the one factor structure as the sample size was inadequate. There were no floor or ceiling effects.

**Conclusions:**

The SFI-Pr PRO can be applied as a specific whole-spine status assessment instrument for clinical and research studies in Persian language populations.

## Background

Spinal pain is an extremely common complaint in the general adult population [1, 2]. The lifetime prevalence for neck and low back pain, which both affect the rates of disability and sick leave [3], have been reported at 48.5% [4] and 70% [5] respectively. In relation to this high prevalence, studies have often focused on neck and low back regions and less on the thoracic or upper back [6] and minimally on the whole-spine as a single kinetic chain. Spinal disorders result in restricted movements [3, 7], functional limitations [5, 7, 8], disability [9–11], reduced health related quality of life and a reduced capacity in the activities of daily living (ADL) [7].

There are various instruments and methods to evaluate spinal health, functional status and the effects of interventions and treatment. Traditional procedures, such as physiological parameters of neural conduction velocity [12], range of motion, muscular strength, endurance [12, 13] and neurological tests [5, 6, 14] have been used. But in many cases these physical parameters are unable to predict the performance of, and effects on ADL [13]. Consequently such traditional methods are less representative of functional status [15]. By contrast, a patient’s participation in their evaluation process using other instruments, such as patient reported outcome (PRO) measures, can lead to a clearer view of functional ability and the effectiveness of any interventions [15] and the individual overall status [9].

The use of PRO instruments falls into five categories of which the initial three apply to all health settings [16, 17] and a further two that are more specific to musculoskeletal situations [16, 18, 19]. The initial three include: i) *generic* - designed to ‘… measure aspects of health status and quality of life which are common to most patients’ [17] and can be used in any condition regardless of diagnosis (e.g. the EQ-5D and SF-36); ii) *condition-specific* - that apply to ‘…a sector … service or … population segment’ [17] (e.g. the Swiss *Spinal* Stenosis Questionnaire); and iii) *disease specific* - such as for cancer (e.g. the Core *Outcome Measures* Index and the Modified McCormick Scale). The final two PRO circumstances include: iv) *regional* - which measure the spine as a single kinetic chain [20] and account for the cervical, thoracic, lumbar and sacral components [e.g. the Spine Functional Index (SFI) and Functional Rating Index (FRI)]; and v) *joint-specific* - which measure a component of the regional kinetic chain [21] (e.g. the Oswestry Disability Index, (ODI) and Roland Morris Questionnaire (RMQ) for the lumbar region and the Neck Disability Index (NDI) for the cervical). Employing regional instruments can result in smaller sample sizes due to improved sensitivity and consequently reduce research time frames [20]. Also costs are lower as these PROs are simpler to use and require reduced administrative burden [18, 19]. The consequences for research and general clinical application are more appropriate and feasible applications [6, 22].

Currently there are least 58 instruments developed to assess spinal status [18, 23, 24]. Among them, the RMQ [25, 26] and ODI [25, 27] are used most commonly for the lumbar spine, and the NDI [28, 29] for the cervical spine. These three PROs account for the greater majority of all spine research PRO results [30, 31], have the highest number of cross-cultural adaptations, and consequently are the most common PRO’s reported in the spine specific literature due to their use in different settings. However, all three have been critically appraised as having flaws in the psychometric structure and practicality. The RMQ as it is a dichotomous response option and consequently fails to allow for a mid-point in cognitive self-recognition [9]; the ODI [32] and NDI [28] due respectively to issues of practicality and borderline suitability of the factor structure [28, 32].

The RMQ, ODI and NDI have all had psychometric characteristics investigated in Persian cultural settings and published in Persian [3, 13]. However, assessment of these published Persian PRO measures suggests deficiencies in: the standardized methodology of tool development [33]; a lack of practicality for evaluating each region of the spinal column within a single kinetic chain concept; no independent validation for the whole spine as a single kinetic unit; and no clarification that a single summated score is validated through the use of a minimum of exploratory factor analysis (EFA) [34]. The only available questionnaires for evaluation of the entire spine are the Bournemouth Questionnaire [35, 36], the FRI [37] and SFI [9] with all being reported as suitable one-factor tools under EFA that ensures each can provide a single summated score [38, 39]. The SFI can be applied in both clinical and research fields [6] and is shown to be both valid and reliable in English [9], Spanish, Chinese, Korean and Turkish [6, 22, 23, 40]. The SFI has also been translated into several other languages that have yet to be published.

The aim of this study was cross-cultural adaptation of the SFI to Persian (SFI-Pr) and determining its psychometric features including validity, reliability, factor structure, standard error of measurement (SEM) and internal consistency in patients suffering spinal disorders. The psychometric characteristics of the SFI-Pr can be compared with the original SFI, other language versions and other spine specific PRO measures, either regional or joint-specific.

## Methods

### Participants

A total of 224 (104 female and 120 male, aged between 18 and 60 years) native Persian speaking patients with spine symptoms referred to three physical therapy clinics by a medical practitioner participated were recruited to this study. Inclusion criteria were neck or back injury of mechanical or degenerative natures diagnosed by a medical practitioner. Exclusion criteria were refusal to participate in the study, LBP as a result of a specific spinal disease (except osteoporosis or osteoarthritis), infection, inflammatory conditions such as ankylosing spondylitis, tumor, fracture or the presence of cauda equina syndrome, age below 18 years, and poor Persian language comprehension. The ethics committee of the University of Social Welfare and Rehabilitation Sciences (USWR) approved the study (No 1395.26). After explaining the aim of the study to the participants, a written informed consent was gained.

### Measures/ questionnaires

#### The spine functional index (SFI)

The SFI was used for cross-cultural adaptation in this research. The SFI is a single factor structure PRO measure with 25-item related to health and quality of life status, functional capacity and ADL [9]. It was developed according to the World Health Organization Standards and derived from the International Classification of Functioning [41]. It has a 3-point response option of Yes’, ‘Partly’ and ‘No’, takes less than a minute to complete and provides information about the patient’s functional status ‘over the last few days’. The 25 responses are summated, the resultant score multiplied by four then subtracted from 100 to give the patient a functional score relative to their normal status [9]. Up to two missing responses are permitted. The Persian (Iranian) version of the RMQ [13] and NDI [3] were also applied to test convergent validity.

*The Neck Disability Index (NDI)*: the NDI PRO measure is used to assess neck functional status [28]. It comprises 10 self-reported items related to pain, ADL and concentration, each rated on a 6-point Likert scale with a final score range of 0 (no disability) to 50 (major disability) which can be expressed as a percentage of disability when multiplied by two. The reliability of the Persian version is reported at ICC = 0.97 [3]. The correlations between the NDI score and the subscales of the SF-36 range from 0.36 to 0.70. A good correlation between the VAS and NDI (0.71) was also reported [13].

#### The Roland Morris Questionnaire (RMQ)

The RMQ is a single page, 24-item dichotomous (Yes/No response format) PRO measure used to assess low back functional status with a total score from 0 (lowest possible) to 24 (highest possible). The Persian version showed excellent test-retest reliability (ICC = 0.86) and validity in low back pain (LBP) patients. The correlation between the RDQ and physical functioning scales of the SF-36 and VAS was 0.62 and 0.36, respectively reported [13].

### Translation and cross-cultural adaptation

The cross-cultural adaptation and translation of the English version of SFI into Persian was conducted according to published guideline [42]. Two independent native Persian speakers performed translation of the original English SFI (forward translation). One translator was a physical therapist and aware of the questionnaire concept and the other was not. After discussing discrepancies a consensus was adopted. Two independent and blinded translators performed backward translation. An expert review committee consisting of one physical therapist, one neurosurgeon, one ergonomist, one psychometrician, all of the translators, and the authors produced a pre-final version of the SFI-Pr.

### Face validity test of the pre-final version

A total of 35 patients with spine disorders (20 males and 15 Females, mean age 34.05 ± 8.57 years) completed the pre-final SFI-Pr in order to test the alternative wording and to check understandability, interpretation, and cultural relevance of the translation. Participants found the questionnaire easy to understand and consequently the SFI-Pr questionnaire was established.

### Statistics

Distribution and normality of the SFI, RMQ, and NDI were determined by the one sample Kolmogorov-Smirnov (KS) test (significance> 0.05). Test-retest reliability was performed using the Intraclass Correlation Coefficient type 2,1 (ICC_2,1_) in a randomly selected sub-sample of *n* = 31 recorded at baseline and repeated, dependent on participant availability, between 3 and 7 days following a period of non-treatment. When alpha and power are fixed at 0.05 and lower than 80% respectively, a minimum sample size of 22 is sufficient to detect the value of 0.50 for the ICC_2,1_. Allowing for an additional 20% attrition rate the sample size required would be 28 [43]. A value above 0.8 was considered evidence of excellent reliability [44].

Internal consistency was assessed using Cronbach’s-α. Its value between 0.70 and 0.95 is considered high with values over 0.95 considered excessive and suggestive of redundancy and potential non-validity [45, 46]. Convergent validity was determined by calculating the Pearson’s correlation between the SFI-Pr and the Persian RMQ and NDI. A minimum correlation of *r* ≥ 0.4 is considered satisfactory (*r* ≥ 0.81–1.0 as excellent, 0.61–.080 very good, 0.41–0.60 good, 0.21–0.40 fair, and 0–0.20 poor) [37]. Participants completed all PRO measures simultaneously.

Factor structure was analyzed using EFA with loading suppression at 0.3 for maximum likelihood extraction (MLE) [46]. The factor extraction had three a-priori requirements: 1) scree plot inflexion; 2) Eigenvalue > 1.0; and variance > 10% [34]. The confirmatory factor analysis (CFA) was conducted on the full 25-items where a best-fit model should present a non-significant chi-square result and the following indices: (1) a Satorra–Bentler scaled chi-square (S-Bχ 2)/degrees of freedom ratio (CMIN/DF) of 2.0 or less; (2) a non-normed fit index (NNFI) no less than 0.90; (3) a Robust-Comparative fit index (Robust-CFI) no less than 0.90; (4) a goodness-of-fit index (GFI) no less than 0.90; and (5) a low root mean square error of approximation (RMSEA) no less than 0.08 [34, 47].

The minimum detectable change at the 90% level (MDC_90_) [48] analysis was used to determine the sensitivity or error score of the questionnaire. The MDC is the reliable change or smallest real difference that reflects true change rather than measurement error. It was calculated by determining the standard error of the measurement (SEM) for the SFI. The SEM was calculated using the formula of [SD$\sqrt{1-r\ }$], where SD is the standard deviation of the measurement and r the test-retest reliability coefficient. Therefore MDC was calculated from [MDC_90_ = SEM$\ast 1.96\ \sqrt{2}$] [49, 50].

Floor and Ceiling effects were calculated by the percentage frequency of the highest and lowest score achieved by participants. If more than 15% of the participants achieve this score, then ceiling and floor effects were considered present [45]. All statistical analysis were calculated using the statistical package for social science version 16 (SPSS 16) for windows and the factorial analysis was done using AIMOS (18version) software. The level of significance was set at *p* < 0.05.

## Results

### Samples characteristics

A total of 224 patients (mean age = 38.8 ± 10.9 years) suffering from neck pain (*n* = 112), thoracic pain (*n* = 13), low back pain (*n* = 87) or multi-region pain (*n* = 12) participated in this study. Of these, a sub-sample (*n* = 31, female = 38.7%) were randomly selected to participate in the test-retest analysis. Demographic characteristics of the study sample are reported in Table 1. The normative mean and standard deviation values for SFI-Pr score were determined (10.15 ± 4.15 point). Also the Item total correlation (Table 2) is presented and includes additional columns for the EFA Communalities, both initial and extracted.

**Table 1:** Demographic characteristics of the participants

Variable		Number (%)	Mean (SD) of age (year)
Age (year)		224	38.76 (10.87)
Gender	Male	120 (53.6)	40.09 (11.13)
	Female	104 (46.4)	37.24 (10.04)
Level of Education	High school	42 (18.8)	–
	Associate’s Degree	95 (42.4)	–
	Bachelor and more	87 (38.8)	–
Site of pain	Cervical	112 (50%)	40.9
	Thoracic	13 (5.81%)	37.3
	Lumbar	87 (38.83%)	41.4
	Multi-region	12 (5.36%)	35.8

*SD* Standard Deviation

**Table 2:** Internal consistency item-total correlation; and EFA Communalities

Items	Internal consistency C-Alpha	EFA Communalities	
	Corrected Item-Total correlation	Initial	Extraction
s1	.279	.330	.253
s2	.338	.342	.999
s3	.067	.179	.145
s4	.352	.442	.999
s5	.346	.395	.375
s6	.463	.393	.336
s7	.343	.261	.197
s8	.296	.210	.111
s9	.419	.376	.490
s10	.574	.481	.600
s11	.495	.398	.385
s12	.460	.454	.542
s13	.528	.527	.651
s14	.511	.475	.483
s15	.455	.421	.394
s16	.495	.384	.360
s17	.521	.537	.666
s18	.526	.540	.638
s19	.495	.464	.543
s20	.483	.448	.505
s21	.553	.511	.573
s22	.468	.414	.339
s23	.389	.458	.524
s24	.517	.450	.499
s25	.581	.541	.612

### Translation process and cultural adaptation

There was no major difficulty in completing the forward and backward translation which corresponded to the original version. Minor modifications were applied in the text based upon cultural relevance. All patients reported no problems or difficulties in completing the SFI. Moreover, there was no missing data and all items were responded to.

### Floor and ceiling effects

None of the subjects achieved the lowest or highest score of the Persian SFI or in excess of 15% floor and ceiling values.

### Internal consistency

Cronbach’s-α value was achieved at 0.80 with individual item ranges of 0.78 to 0.82 indicating a high level of internal consistency.

### Tests-retest reliability

A total of 31 patients completed the SFI questionnaire twice with an interval of 3–7 days, being a period of non-treatment. There was no significant difference between test and retest means scores. The high ICC value (0.96) with an individual range of 0.83 to 0.98 indicated excellent test-retest reliability.

### Measurement error

Measurement error from the SEM and MDC were respectively 2.52 and 4.58%.

### Convergent validity

Convergent validity between the SFI and RMQ was high (*r* = 0.69), and moderate between the SFI and NDI (*r* = 0.57).

### Factor structure

**Fig. 1:**
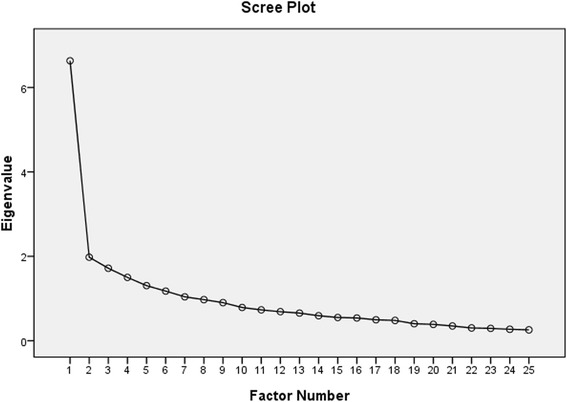
The scree plot supported a one-factor solution

The EFA using MLE was conducted on the 25 items. The Kaiser-Meyer-Olkin (KMO) measure which was found at 0.83 was well above the acceptable limit of 0.5 [51] and verified the sampling adequacy for the analysis. Bartletts’s test of Sphericity [x^2^(300) = 185,425.08, *p* < 0.001] indicated that correlations between items were sufficiently large for factorial analysis. In an initial analysis, Eigenvalues for seven factors were > 1, however only one factor accounted for more than 10% variance (26.53%). Further and the scree plot inflexion distinctly occurred at the second point (Fig. 1). Together, these three criteria suggested a one-factor structure was most likely. The factor loading for the one factor solution is shown in Table 3. An independent blind analysis by separate bio-statisticians of these findings concluded that on the basis of parsimony and the available sample size, a one-factor structure was the most likely.

**Table 3:** Factor loading items for the one-factor solution and average score of items

Item	Explanation	Factor loading	Item average score^a^
1	Stay at home	0.222	0.3616
2	Change position	0.364	0.5848
3	Avoid heavy activities	0.072	0.4933
4	Rest most of the time	0.357	0.3103
5	Ask others to do things	0.380	0.2567
6	Often feel pain or discomfort	0.535	0.5089
7	Difficulty in carrying and lifting	0.396	0.4933
8	Different Appetite	0.354	0.3438
9	Walking, normal recreational and sport activities	0.501	0.4442
10	Difficulty doing family and household duties	0.632	0.4129
11	Do not sleep well	0.571	0.4330
12	Need help in self-care	0.535	0.2701
13	Routine daily activity	0.597	0.3348
14	More irritable and grumpy	0.580	0.3661
15	Feel weaker and stiffer	0.519	0.4621
16	Independency in transportations	0.556	0.3326
17	Get dressed more slowly	0.610	0.2656
18	Difficulty moving in bed	0.625	0.3973
19	Difficulty reading /focusing	0.583	0.4129
20	My seating has been affected	0.573	0.4509
21	Difficulty sitting on chairs then standing up	0.640	0.4107
22	Stand for a short time	0.541	0.4554
23	Difficulty in squatting/ kneeling	0.467	0.4933
24	Difficulty reaching down	0.594	0.4330
25	Go upstairs more slowly than usual or using hand rail	0.654	0.4286

^a^It is the average score for each item of the SFI

The CFA was inconclusive as only the RMSEA test was within the minimum required defined parameters, though the remaining four parameters approached the minimums where CMIN/DF = 2.5, NNFI = 0.652, CFI = 0.752 and GFI = 0.798. Consequently, in view of the inadequate sample size and four parameters that approach but are not above the required cutoffs, the factor structure under CFA cannot be either confirmed or negated by the current findings.

## Discussion

The purpose of this study was to translate and cross-culturally adapt the original SFI questionnaire from English to Persian and test psychometric properties. In order to maintain the content validity of an instrument at a conceptual level across different countries and cultures, the items must not only be well translated linguistically, but also adapted culturally [33, 52, 53]. During this phase, most patients completed the questionnaire unaided, without difficulty and there was no lack of clarity. Some minor modifications in translation were performed for cultural reasons. In section one, questions number #3 and #7, the weight measurement unit of pounds (lbs) is unfamiliar with Persian society. Consequently 10lbs was omitted and just the System International kilogram unit for weight (kg) was maintained.

Considered psychometrics properties in this study were reliability and validity. Internal consistency, test-retest reliability and measurement error are the critical properties in the reliability domain. Convergent and construct validity are predominant in the validity domain. It was shown that the SFI-Pr had very high test-retest reliability (ICC_2.1_ = 0.96) that was identical to the Spanish and Chinese versions (ICC_2.1_ = 0.96) [22], very close to the original English (ICC_2.1_ = 0.97) [9], but higher than both the Turkish [6] and Korean [23] (ICC_2.1_ = 0.93). Further, the internal consistency (α = 0.80) was lower than the four previously reported versions including the original (α = 0.91) [9], Chinese (α = 0.91) [40], Turkish and Korean (α = 0.85) [6] and Spanish (α = 0.84) [22] but above the required threshold [45] for acceptance.

The SFI-Pr demonstrated lower error values (SEM = 2.52% and MDC_90_ = 4.58%) in comparison to all previous reported studies [6, 9, 22]. These lower values allow for improved sensitivity in detecting assessment results or treatment effectiveness and change over time. This potentially could be related to the comparably lower α value or a low variation in the SD of baseline presenting scores. The absence of floor and ceiling effects concluded with the sensitivity results, and assists detecting any changes after interventions and assessment.

Evaluating the convergent validity with the NDI and RMQ showed a high correlation with the RMQ (*r* = 0.69) and moderate correlation with the NDI (*r* = 0.57). For the lumbar portion, this is lower than the Spanish (*r* = 0.79) and Korean (*r* = 0.75) findings for the RMQ [22, 23]. In the Turkish and Chinese studies the ODI replaced the RMQ where correlation was *r* = 0.71 [6] and r = 0.75 [40] respectively. High correlation between the Persian ODI and RMQ has been shown (r = 0.71) [13], consequently our results can be indirectly compared with the previous studies [6, 22].

For the cervical portion, the correlation between the SFI-Pr and NDI (r = 0.57) was similar to the Korean (*r* = 0.53) [23], Turkish (*r* = 0.58) and Chinese (*r* = 0.61) SFI findings, but higher than the Spanish (*r* = 0.46). These differences may be attributed to the diverse cultural and geographical features of the selected participants. The Korean study also used the FRI with a correlation of r = 0.57 [23], which was substantially lower than the *r* = 0.87 found in the original English version. Further, in an Iranian population the sample is effectively mono-cultural with participants being predominantly of Persian background. In the Spanish, and to a lesser degree in the Turkish, Korean and Chinese studies, the potential for individuals of a more diverse cultural background, as well as language and population diversity may be present but is not indicated, which may affect the findings. This cultural diversity is particularly high for the original Australian study where participants are from a multi-cultural society with significant variation in cultural background and ethnicity that together made up the representative sample. It has been noted in the literature that factors such as sample size, characteristics and the stage of disease or problem of the individual patients may affect the results of a Pearson correlation coefficient [54, 55].

Our subjects were approximately 10 years younger than those in the original, Turkish, Korean and Spanish SFI studies. The mean age is not reported in the Chinese study. Further, male participants in particular were lower than the Turkish and Spanish studies but higher than the Korean. Also the distribution of the subjects in terms of the involved region was marginally different, but this is unlikely to have affected the findings. The cervical representation at 50% was higher but comparable to the previous ranges of 30–47%; thoracic, at 6%, was comparable to the Spanish at 4%, Korean at 3%, Turkish at 1% and Chinese at 0%, but notably lower than the 24% in the original; lumbar was 10–14% lower at 39% compared to the range of 49–53%; and the multi-area representation was comparable to the Spanish at 6%, Chinese at 4% and Turkish at 1%, but notably lower than the 13% in the Korean study and 23% in the original.

The construct validity of the SFI questionnaire was tested with EFA. The single factor solution was found in all four previous analysis of the SFI [6, 9, 22, 40], however it was suggested that as some factors were notably below the loading suppression cutoff of 0.30 some items could potentially be removed. Consequently, item redundancy may be present and a shortened tool should be considered [6]. This recommendation is also supported by this study as the Iranian culture, particularly for those with a lower level of education and broad scientific and health knowledge, usually underestimate the impact their condition can have. This may lead to a failure to understand the initial management aspect in relation to their health status and work for a LBP or neck problem. Consequently responses to times #1 ‘I stay at home more’ and #3 ‘I avoid heavy jobs’ could be affected by this social cultural contributor. However from the perspective of parsimony and in accordance with the a-priori requirements, the single factor structure is supported.

The Chinese, Spanish, and Turkish versions [6, 22, 40] found the dominant factor accounted for respectively 32, 27.4 and 24.2% of variance. However, in each study, as in this study, only one factor had variance of > 10%. In this study, the variance level (26.5%) was very close to that found in the Spanish and Turkish versions [6, 22], though lower than in the original and Chinese (33.4%) [9]. It was 4–6 times higher than any of the other factors, none of which exceeded 10%. The scree plot inflexion criterion remains a subjective assessment but occurred distinctly at the second data factor; therefore, supporting the one factor structure from the perspective of parsimony and tradition.

The CFA, in a substantially limited population and using the same sample as the EFA, found only the one parameter of the five above the threshold, though the remaining four approached the required minimums. The CFA findings from our study were slightly better than those in the Chinese study where CFA was also performed, despite their small sample of *n* = 271. In both studies RMSEA was the only parameter, of the five, that supported an excellent single factor structure. However, as CFA determines whether the structure is multi-faceted or unitary, these results can state that the structure is not an ideal fit for a one factor solution. However, there is an inadequate sample size and the remaining four parameters approached the required cutoffs and may have been significant in an appropriately powered analysis. Consequently, the one factor solution cannot be either confirmed or negated by the current CFA findings, particularly in view of the statistical limitations. Similarly, further analysis on a shortened version of the SFI will be necessary and indicated as currently under publication submission.

### Study limitations and strengths

One limitation of this study was only the EFA essentially determined the SFI dimensional structure with sample size being inhibitory of appropriate CFA. The EFA helps obtain preliminary information about the dimensionality. With only four previous SFI- EFA studies, the available supporting research is low in this regard. By contrast, clarification of the status of the factor structure is usually done using CFA. It is suggested a sample size of at least 5–10 times greater than the EFA be used [6], which was beyond the scope of this study. Consideration of Rasch Analysis could also be made. However it is noted that Rasch Analysis and Factor Analysis are distinctly different [34]. Rasch analysis indicates equal informativeness between items to create a single “true” score. By contrast, CFA uses different assumptions, modeling and estimations to determine whether the structure is multi-faceted or unitary. Rasch Analysis was beyond the scope of this study as the population sample was insufficient and it was not part of the original aims.

A further study limitation was longitudinally. Ongoing data measurement was impossible due to the time restraints and ethics obligations of the study, making it cross-sectional only. Further, generalizability of the results is limited as patients were only selected from physiotherapy centers and not the general population, spine clinics or specific tertiary, surgical or inpatient sources.

The study strengths include the use of the standard methods in translation and cultural adaptation and psychometrics assessment of the SFI-Pr. This consequently expands the available specific number of PRO measures for Persian speaking patients and professions.

## Conclusions

To our knowledge, this developed Persian version of the SFI (SFI-Pr) is the only whole spine outcome measure available in Iran and for Persian speakers. The results demonstrated it is possible to translate this questionnaire into Persian without loss of the original psychometric properties. Consequently, the SFI-Pr can be applied as a specific whole spine status assessment instrument for clinical and research studies in Persian language populations, however further research is necessary in larger population samples to clarify the factor structure through CFA and possibly Rasch analysis.

## Abbreviations

ADL: Activities of daily living
DF: Degrees of freedom
EFA and CFA: Exploratory and confirmatory factorial analysis
FRI: Functional rating index
GFI: Goodness of fit index
ICC: Intraclass correlation coefficient
KMO: Kaiser-Meyer-Olkin
KS: Kolmogorov-Smirnov
LBP: Low back pain
MDC: Minimum detectable change
MLE: Maximum likelihood extraction
NDI: Neck disability index
NNFI: Non-normed fit index
ODI: Oswestry disability index
PRO: Patient reported outcome
QDS: Quebec back pain disability scale
RMQ: Roland-morris disability questionnaire
RMSEA: Root mean square of approximation
SD: Standard deviation
SEM: Standard error of measurement
SFI: Spine functional index
SFI-Pr: SFI for Persian-speaking patients
USWR: University of social welfare and rehabilitation sciences
